# The effect of Iran’s health sector evolution plan on hospitals performance indicators: an interrupted time series analysis

**DOI:** 10.3389/frhs.2023.1144685

**Published:** 2023-08-21

**Authors:** Shahin Soltani, Satar Rezaei, Ali Kazemi-Karyani, Jila Azimi, Faramarz Jalili, Bahman Roshani, Farid Najafi, Parnia Bagheri, Yahya Salimi

**Affiliations:** ^1^ Research Center for Environmental Determinants of Health (RCEDH), Health Institute, Kermanshah University of Medical Sciences, Kermanshah, Iran; ^2^ Department of Epidemiology, School of Health, Kermanshah University of Medical Sciences, Kermanshah, Iran; ^3^ School of Health Administration, Dalhousie University, Halifax, NS, Canada; ^4^ Department of Anesthesiology, Imam Reza Hospital Center, Kermanshah University of Medical Sciences, Kermanshah, Iran; ^5^ Social Development and Health Promotion Research Center, Health Institute, Kermanshah University of Medical Sciences, Kermanshah, Iran

**Keywords:** hospitalization, hospital, health sector evolution plan, interrupted time series, Iran

## Abstract

**Background:**

The Health Sector Evolution Plan (HSEP) was set up in Iran’s health system to respond to some of the main problems in hospitals and other health sectors. We aimed to compare the effect of the HSEP on teaching hospital performance before and after the implementation of the HSEP through the interrupted time series (ITS) analysis.

**Methods:**

With a cross-sectional design, data collection was performed in 17 teaching hospitals affiliated with the Kermanshah University of Medical Sciences (KUMS). We used the existing data on three indicators of hospitalization rate (per 10,000 population), Emergency Department Visits (EDVs) (per 10,000 population), and in-hospital mortality (per 10,000 population). The monthly data from 2009 to 2019 was analyzed by the ITS method 60 months before and 61 months after the HSEP.

**Results:**

We found a non-statistically significant decrease in the monthly trend of hospitalization rate relative to the period before the HSEP implementation (−0.084 per 10,000 population [95%CI: −0.269, 0.101](. There was a statistically significant increase in the monthly trend of EDVs rate compared to before the HSEP implementation (1.07 per 10,000 population [95%CI: 0.14, 2.01]). Also, a significant decrease in the monthly trend of in-hospital mortality compared to before the HSEP implementation [−0.003 per 10,000 population (95%CI: −0.006, −0.001)] was observed.

**Conclusion:**

Our study demonstrated a significant increasing and decreasing trend for EDVs and in-hospital mortality following the HSEP implementation, respectively. Regarding the increase in hospitalization rate and EDVs after the implementation of HESP, it seems that there is a need to increase investment in healthcare and improve healthcare infrastructure, human resources-related indicators, and the quality of healthcare.

## Introduction

In 2014, a series of reforms called the Health Sector Evolution Plan (HSEP) was implemented in Iran’s health system to respond to some of the main problems in its performance. Largely, HSEP was in line with the third 5-year health development national strategies (2011–2016) and the new government’s commitments to achieve universal health services coverage.

This stepwise national plan approved by the cabinet on April 30, 2014, to promote equitable access to healthcare and to improve the quality of hospital care included eight main packages of healthcare services (1): (i) increasing healthcare coverage, especially in remote and rural areas, and recruiting physicians, healthcare workers, and personnel in underserved areas; (ii)reducing out-of-pocket expenses; (iii) improving the quality of outpatient visits; (iv) providing specialist doctors in hospitals; (v) improving the quality of accommodation services, (vi) promoting natural childbirth; (vii) ensuring financial protection for diseases with long and expensive treatments; (viii) changing health tariffs to reduce informal payments and promote cost-effective interventions, and (ix) building ambulance helicopter base centers. This plan was estimated to provide health services for about 9–10 million people in marginalized areas of Iran (2).

To date, various studies have been conducted to evaluate the effect of HSEP on teaching hospitals in Iran. On the one hand, some studies show that HSEP has not had a significant effect on the performance of hospitals in Iran. For example, in the study by Goudarzi et al. (2021), in Kerman, there was no significant increase in university hospitals’ efficiency and productivity following HSEP implementation (3). Hashemipour et al. in a systematic review found that despite the expensive cost of HSEP and the relative satisfaction of patients, the government had not met all the demands of nurses and some physicians (4). On the other hand, some indicators show that HSEP has had a significant effect on the performance of teaching hospitals. For example, a study by Shamsaei et al. (2021), in Zabol, indicated that the implementation of HSEP has increased the average length of stay index, the ratio of beds to be fixed, and the number of injuries in the hospitals studied (5). Beiranvand et al., in Lorestan province, found that HSEP has resulted in a significant increase in the hospitalization rate (1). Furthermore, some studies show a decrease in out-of-pocket payment(OOP) for inpatient services (6), higher satisfaction of hospitalized patients with the services provided by HSEP (4), and a higher quality of inpatient services after the its implementation (7).

Regarding the lack of sufficient evidence in the Kermanshah province, we aimed to compare the effect ofHSEP on teaching hospital performance before and after its implementation through the interrupted time series analysis. Given the existing data, we applied three indicators of hospitalization rate (per 10,000 population), EDVs(per 10,000 population), and in-hospital mortality (per 10,000 population) from 2009 to 2019.

## Materials and method

### Study setting and data collection

Our cross-sectional study was conducted in the Kermanshah province, located in the western part of Iran, with a population of about 2 million people. Data collection was performed in 17 teaching hospitals affiliated with the Kermanshah University of Medical Sciences (KUMS). In this study, we used the existing data on three indicators of hospitalization rate (per 10,000 population), EDVs(per 10,000 population), and in-hospital mortality (per 10,000 population). We extracted the population data from the Iranian Statistical Center (ISC).

### Statistical analyses

The current study aimed to examine the effect of HSEP on hospitalization rate, EDVs, and death rate in the 17 hospitals affiliated with the Ministry of Health and Medical Education (MOHME) in the Kermanshah province. The monthly data from 2009 to 2019 was analyzed by the ITS method 60 months before and 61 months after HSEP implementation.

Data were summarized and reported with mean standard deviation for quantitative variables. The ITS design was applied for modeling the effect of HSEP on the main variables of the study, including hospitalization, EDVs, and death rate. In this analysis, the effect of a large-scale intervention on the explanatory variables was investigated in the form of a quasi-experimental study. This study aimed to determine whether this intervention has changed the trend of hospitalization rate, EDVs, and deaths rate overtime or not. In this analysis, three effects were examined: the pre-intervention slope (β_1_), change in slope (β_2_), and change in trend (β_3_). The Newey-West technique and the assumption of independence were applied for fitting the model, and the Cumby-Huizinga autocorrelation test respectively. We evaluated autocorrelation in different lags. If the autocorrelation is significant at a specific lag, this lag of autocorrelation in the ITS model should be introduced for establishing this pre-assumption. Accordingly, the model was fitted with lag1 firstly and then the Cumby-Huizinga autocorrelation test was performed. Also, a significant step was selected for entering the model in the next step.

To present the findings, coefficients and their 95% confidence intervals with associated *P*-values were presented for the *β*_1_, *β*_2_, and *β*_3_. We showed the ITS graphs for each variable separately. We used Stata 14 software to analyze data at a 0.05 significance level.

To estimate the ITS model for each indicator, a regression model was estimated as follows:

$$Y_{t}=\beta_{0}+\beta_{1}\mathrm{Tt}+\beta_{2}\mathrm{Xt}+\beta_{3}X_{t}T_{t}+\varepsilon_{t}$$

*Y_t_*: the value of each indicator per month.
*X_t_*: intervention.
*X_t_T_t_*: the interaction of time and intervention.
*β*_1_: the time trend of the indicator without considering the intervention.
*β*_2_: The immediate effect of the intervention (the immediate effect of the intervention in the month after the intervention) on the desired indicators shows the changes in the level of the indicator due to the intervention.
*β*_3_: the continuous effect of the intervention on the desired indicators as the change of the indicator trend due to the intervention.
ε_t_: error.

## Results

The mean of hospitalization was 64.93(SD = 4.09) and 82.94(SD = 6.39) per 10,000 population before and after HSEP implementation, respectively. There was an increase in the mean of EDVs after the implementation of HSEP of 574.05(SD = 81.11), compared to the rate before HSEP of 403.78(SD = 51.55) per 10,000 population. The mean of in-hospital mortality was calculated at 0.54 (SD = 0.08) and 0.49 (SD = 0.08) before and after HSEP, respectively.

### The effect of HSEP on the hospitalization rate

**Figure 1:**
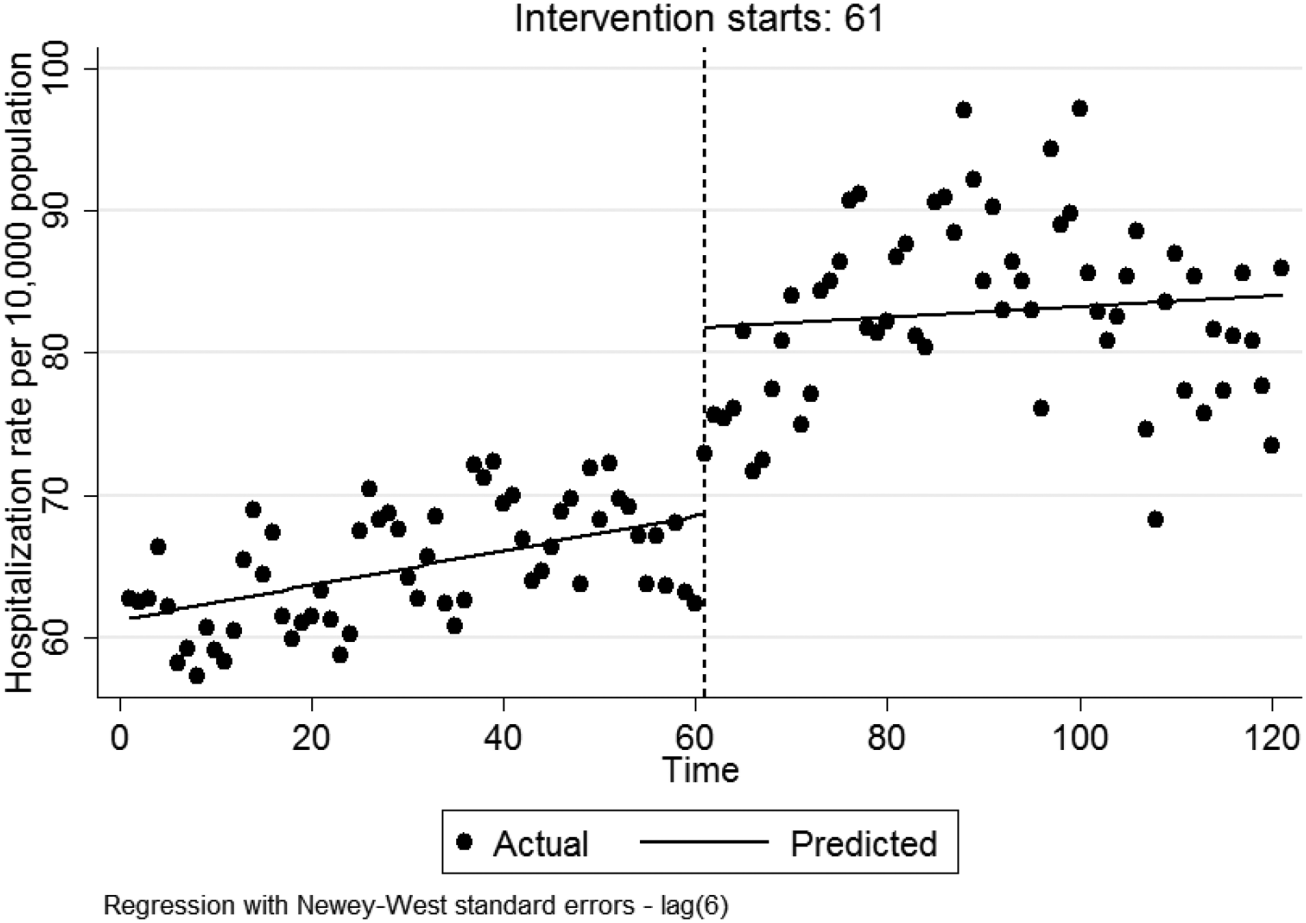
The effect of HSEP on the hospitalization rate after the intervention.

Regarding Table 1, in the first month following HSEP implementation, a significant increase in the hospitalization rate was observed by 13.152 per 10,000 population (95%CI:7.269, 19.036). There was a non-statistically significant decrease in the monthly trend of hospitalization rate relative to the period before HSEP implementation (*β*_3_ = −0.084 per 10,000 population [95%CI: −0.269, 0.101](. After HSEP implementation, the trend of hospitalization rate insignificantly increased on average by 0.037 per 10,000 population per month (95%CI: −0.117, 0.193). Figure 1 shows the effect of HSEP on the hospitalization rate after the intervention.

**Table 1:** Interrupted time series analysis to evaluate the effect of the HSEP on hospitalization rate after the intervention.

Hospitalization rate	Coefficient	SE^a^	[95% Conf. interval]		*P*-value
			Lower	Upper	
Intercept, *β*_0_	61.220	1.175	58.07	63.547	<0.001
Pre-intervention slope, *β*_1_	0.121	0.037	0.047	0.196	0.001
Change in slope after intervention, *β*_2_	13.152	2.970	7.269	19.036	<0.001
Change in trend after intervention, *β*_3_	−0.084	0.093	−0.269	0.101	0.371
Post-intervention linear trend^b^	0.037	0.078	−0.117	0.193	0.630

^a^ Newey–West standard errors.

^b^ This obtained from the following time trend equation: *Ypt*=*βp*0+*βp*1 ∗ time*pt* + *εt*; where *Ypt* is the value of hospitalization rate at time *t* after the intervention and time*pt* is the time trend variable.

### The effect of HSEP on EDVs

**Figure 2:**
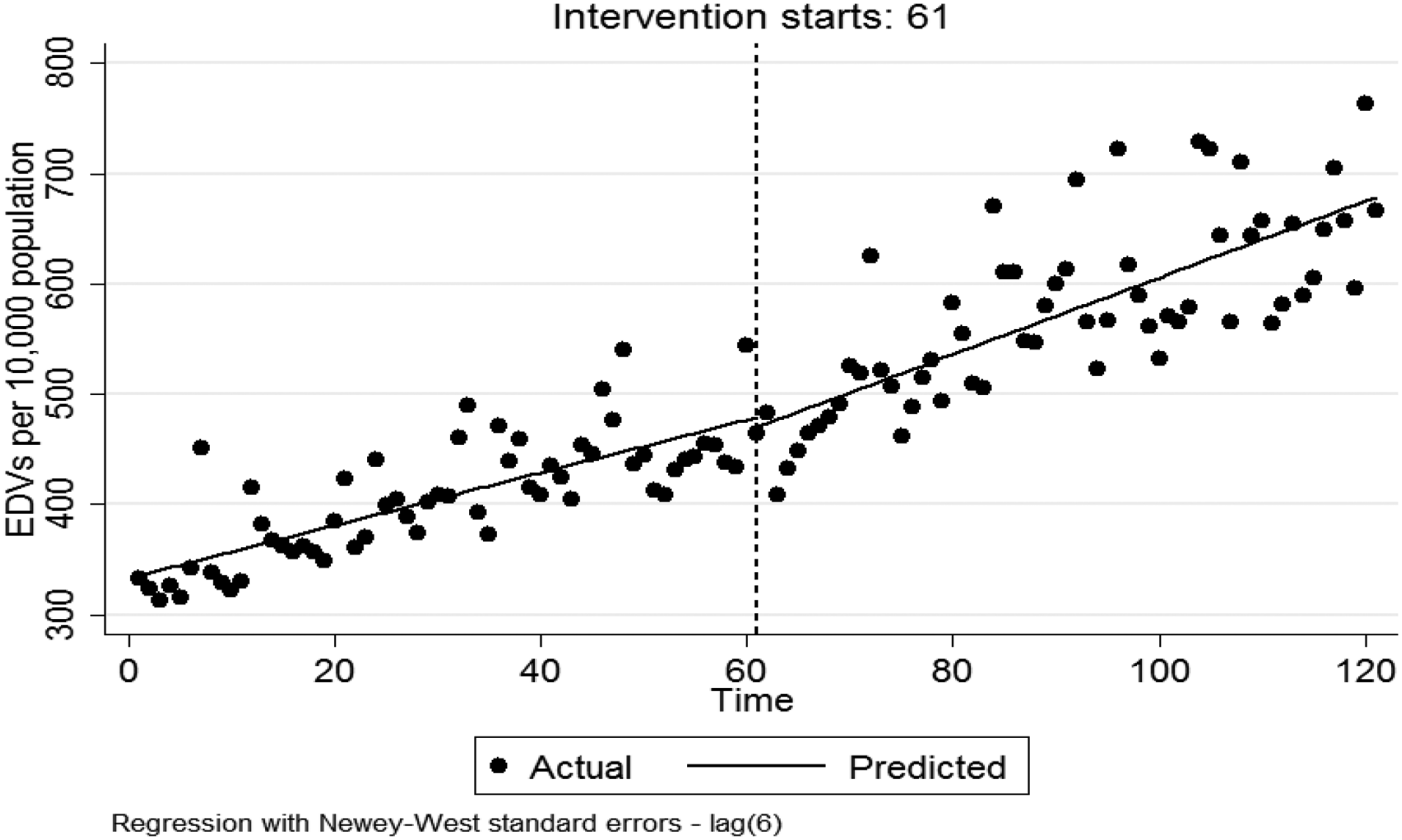
The effect of HSEP on the EDVs rate after the intervention.

As shown in Table 2, in the first month after HSEP implementation, there was a non-significant decrease in EDVs rate [*β*_2_ = −8.863 per 10,000 population (95%CI: −45.052, 27.326)]. There was a statistically significant increase in the monthly trend of EDVs rate compared with before HSEP implementation (*β*_3 _= 1.078 per 10,000 population [95%CI: 0.148, 2.009](. After HSEP implementation, trend of EDVs rate sinficantly increased on average 3.47 per 10,000 population per month (95% CI: 2.696, 4.251). Figure 2 shows the effect of HSEP on EDVs after the intervention.

**Table 2:** Interrupted time series analysis to evaluate the effect of the HSEP on EDVs after the intervention.

Emergency departments visits	Coefficient	SE^a^	[95% Conf. interval]		*P*-value
			Lower	Upper	
Intercept, *β*_0_	333.069	6.595	320.007	346.131	<0.001
Pre-intervention slope, *β*_1_	2.395	0.276	1.848	2.941	<0.001
Change in slope after intervention, *β*_2_	−8.863	18.273	−45.052	27.326	0.629
Change in trend after intervention, *β*_3_	1.078	0.469	0.148	2.009	0.023
Post-intervention linear trend	3.474	0.392	2.696	4.251	<0.001

^a^ Newey–West standard errors.

### The effect of HSEP on in-hospital mortality

**Figure 3:**
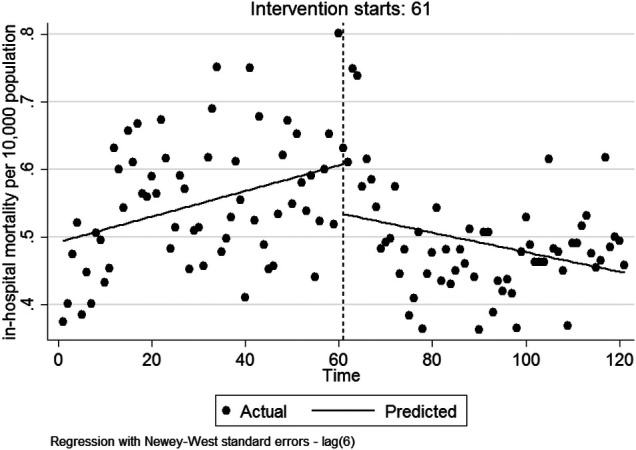
The effect of HSEP on in-hospital mortality after the intervention.

Regarding Table 3, in the first month followingimplementation, there was a non-significant decrease in mortality rate [*β*_2_ = −0.073 per 10,000 population (95%CI: −0.167, 0.019)]. There was a significant decrease in the monthly trend of in-hospital mortality compared to before HSEP implementation [*β*_3_ = −0.003 per 10,000 population (95%CI: −0.006, −0.001)]. After HSEP implementation, the trend of in-hospital mortality, on average, decreased 0.1% per month (95%CI: 0.003, 0.0009). Figure 3 represents the effect of HSEP on in-hospital-mortality after the intervention.

**Table 3:** Interrupted time series analysis to evaluate the effect of the HSEP on in-hospital mortality after the intervention.

In-hospital mortality	Coefficient	SE	[95% Confidence interval]		*P*-value
			Lower	Upper	
Intercept, *β*_0_	0.492	0.363	0.420	0.564	<0.001
Pre-intervention slope, *β*_1_	0.001	0.0008	0.0001	0.003	0.033
Change in slope after intervention, *β*_2_	−0.073	0.047	−0.167	0.019	0.121
Change in Trend after intervention, *β*_3_	−0.003	0.001	−0.006	−0.001	0.034
Post-intervention linear trend	−0.001	0.001	−0.003	0.0009	0.222

## Discussion

The current study aimed to evaluate the effects of HSEP on the performance of teaching hospitals in Kermanshah, Iran.

Our findings showed that HSEP had a positive association with the hospitalization rate in the hospitals included in the study. In other words, the trend of time changes after HSEP implementation compared to before the intervention indicated a significant increase in the hospitalization rate. Similarly, Beiranvand et al. found that in the first month of implementation, the hospitalization rate increased by 2.62. They reported that the hospitalization rate increased by 0.68 compared to the first month after the launch of HSEP (8). Also, a study by Janati et al. indicated that the risk of being readmitted within 30 days after the reform was significantly higher (worse) compared to pre-reform hospitalization (9).

One of the main reasons may be due to decrease in OOP for inpatient visits following implementation. A study by Zarei et al., in Tehran, indicated that the amount of OOP for inpatient visits was 10.2% which has been in line with the goal of HSEP (6). Maharloo et al., in Shiraz, found that the mean OOP after the health transformation plan for cardiovascular patients has decreased significantly from 10,649,295 Rial to 6,971,268 Rial (10).

HSEP in Iran aimed to improve the quality and accessibility of healthcare services in the country, particularly in the public sector. One of the key goals of the HSEP was to increase hospital bed capacity, and as a result, there was a significant increase in the frequencey of hospitalizations in public hospitals in Iran (9, 11).

While the increase in hospitalization rate may be seen as a positive outcome of the HSEP, it has also led to some unintended consequences. For example, the increase in hospitalizations has put a strain on the healthcare system, resulting in overcrowding and long wait times for patients. This has resulted in overcrowding and long wait times for patients. Additionally, this has led to an increase in healthcare costs, which has caused concerns for the sustainability of the plan and equity of financing (12).

Moreover, the increase in indicators like bed occupancy rate may not necessarily be an indicator of improved health outcomes. Hospitalizations may be necessary in some cases, but in others, they may be avoidable through better preventive care, primary healthcare, and disease management. Therefore, it is important to ensure that hospitalizations are appropriate and necessary, and that there are adequate resources and infrastructure to support patients in both inpatient and outpatient settings.

The results of this study indicate that the interaction between intervention and time for EDVs was significant with a positive coefficient. However, literature shows mixed results in Iran. A study by Cheshmekaboodi et al. indicated that implementation of the HESP has had a positive association with EDVs (13). On the contrary, Emamgholipour et al. in Tehran found that implementation of the HSEP had no significant effect on the rate and trend of indices such as EDVs and percentage of patients discharged against medical advice (11).This intervention had a positive effect on the rate and trend of the average response time for emergency tests (14). Regarding this discrepancy in the literature, it could be concluded that the effect of HSEP on emergency department performance is not universal across teaching hospitals in Iran. Factors such as type of hospital (general or specialized hospital), hospital function (teaching hospital, acute care facility, long-term hospital, etc.), hospital size, and the ratio of specialist physicians to health professionals may affect the outcome of HSEP in emergency departments (13–15).

Also, EDVs can be influenced by various factors, such as population demographics, healthcare infrastructure, and health policies. It is possible that the HSEP has had an impact on the rate of ED visits, but further research is needed to estimate the relationship between the two.

By improving access to primary healthcare services and promoting preventive measures, such as vaccination programs, screening tests, and health education interventions, the HSEP may have helped reduce the incidence of preventable illnesses and injuries, and thus the need for emergency care.

However, it is important to note that the increase in hospital bed capacity resulting from the HSEP may have led to more patients being referred to emeregencey departments for non-emergency care, which could contribute to overcrowding and longer wait times for patients in need of urgent care. This could potentially lead to an increase in the rate of EDVs.

Overall, further research is needed to fully understand the relationship between the plan and healthcare utilization trends. Monitoring healthcare utilization trends and evaluating the effectiveness of healthcare policies and interventions is essential to ensure that they are meeting the needs of the population.

The ITS analysis indicated that in-hospital mortality has decreased monthly by about 1% following HSEP. Compared to before implementation of HSEP, we observed a significant decreasing trend of in-hospital mortality, with an average monthly decrease of 3%. This finding shows that the aim of increasing availability, quality, and access to affordable health services in HSEP probably has had a positive effect on in-hospital mortality. For example, Alipour et al. found that HSEP has caused an increase in the economic burden of cardiovascular diseases in the northwest of Iran that was due to the increase of all direct and indirect costs, except the OOP expenditure (16). It should be noted that positive effects of HSEP on hospitals’ performance should not be attributed solely to this intervention. We think that more detailed studies are needed to investigate the effects of HSEP on hospital performance.

Despite positive effects, the HTP faces some serious challenges in Iran’s health system. Mosadeghrad et al. revealed that this plan has doubled the tariff on healthcare services which has imposed a financial burden on Iranian public health insurance companies and made it difficult to finance Iran’s health system (17). Also, the remarkable rise in the rate of inflation, following the Unites States Sanctions against the Central Bank of Iran in 2018, has imposed higher healthcare costs on both patients and the healthcare system in Iran. A sustainable health financing system should be developed to control the costs and provide equitable health services.

Our findings show that HSEP has led to an increase in hospitalization rate and EDVs significantly following its implementation.

## Limitation

In this study, we used existing data from teaching hospitals in Kermanshah. To provide a comprehensive picture, there is a need to compare indicators across all teaching hospitals in Iran. While we investigated the effects of HSEP on teaching hospitals, more studies are needed to evaluate these effects on both teaching and private hospitals in Iran. Given the lack of control for time-varying confounders in the present study, any interpretations of the causal effect of any association should be undertaken with caution.

Furthermore, it is important to consider various factors that may contribute to the effects of HESP on teaching hospitals in Iran, including hospital type (general or specialized hospital), hospital function (teaching hospital, acute care facility, long-term hospital, etc.), hospital size, and the ratio of health professionals (18). Given the potential impact of these factors on hospital performance, we recommend conducting additional studies at the national level to investigate the relationship between these explanatory variables and hospital performance. Such studies could provide valuable insights into the complex interplay of factors that influence the effectiveness of HESP and inform the development of evidence-based policies and interventions to improve hospital performance.

## Conclusion

Our study demonstrated a significant increasing and decreasing trend for EDVs and in-hospital mortality following HSEP implementation, respectively. Our study recommends continuous monitoring of HSEP to identify health-related outcomes in teaching hospitals in Iran. Also, regarding the increase in hospitalization rate and EDVs after the implementation of HESP, it seems that there is a need to increase investment in healthcare, and improve healthcare infrastructure, human resources-related indicators and the quality of healthcare.

## Data Availability

The raw data supporting the conclusions of this article will be made available by the authors, without undue reservation.
